# Physical Examination in Modern Surgical Practice

**DOI:** 10.7759/cureus.110098

**Published:** 2026-06-02

**Authors:** Mohammad Safadi, Amir Farah

**Affiliations:** 1 General Surgery, EMMS Nazareth Hospital, Nazareth, ISR; 2 Surgery, Medical College of Wisconsin, Milwaukee, USA

**Keywords:** health professional's education, imaging modalities, pocus (point of care ultrasound), surgeon physical examination, surgery general, technology enhanced education

## Abstract

Advances in diagnostic imaging, laboratory testing, and point-of-care ultrasound have transformed surgical practice, allowing clinicians to identify pathology with unprecedented speed and accuracy. As these technologies have become increasingly integrated into patient care, the bedside physical examination has assumed a less prominent role in clinical decision-making. This shift is often viewed as a natural consequence of technological progress. However, the decline of the bedside physical examination may reflect more than the availability of superior diagnostic tools. It may also represent medicine’s growing intolerance of uncertainty.

Historically, surgeons routinely made decisions despite incomplete information, relying on history, physical examination, and clinical judgment to guide management. Although modern diagnostics have reduced uncertainty, they have not eliminated it. Imaging studies remain subject to interpretation, clinical presentations remain heterogeneous, and important decisions frequently must be made before definitive answers are available. The physical examination has traditionally served not only as a means of detecting disease but also as a framework for observation, interpretation, and bedside judgment. These skills remain essential even as diagnostic technologies continue to evolve.

The relationship between the declining role of the physical examination and the increasing pursuit of diagnostic certainty in contemporary surgical practice should be re-explored. The enduring value of bedside assessment lies not in competing with advanced diagnostics but in cultivating the clinical judgment required to act when certainty remains elusive. As surgery enters an era increasingly shaped by imaging, artificial intelligence, and data-driven decision support, preserving the ability to evaluate patients directly may be as important as adopting the technologies that continue to redefine care.

## Editorial

A patient arrives in the emergency department with abdominal pain. Before the surgeon enters the room, laboratory studies have been completed, imaging has been completed, and a radiology report is already available. By the time the consultation begins, the diagnosis may be largely established.

Few would argue that this represents a problem. Modern diagnostic technology has transformed surgical care. Computed tomography can identify pathology with a speed and accuracy unimaginable to previous generations [1]. Conditions that once required exploratory surgery to diagnose can now be characterized within minutes. Point-of-care ultrasound (POCUS) places additional diagnostic capability directly at the bedside. These advances have improved patient care and undoubtedly prevented harm.

Yet alongside this progress, another change has occurred. The bedside physical examination occupies a progressively smaller role in clinical decision-making. This shift is usually viewed as an inevitable consequence of technological advancement: more accurate tests have naturally displaced less accurate ones. While there is truth in that explanation, it may not tell the whole story.

The decline of bedside examination may reflect something broader than improved diagnostics. It may reflect medicine's growing intolerance of uncertainty. For most of medical history, uncertainty was unavoidable. Physicians and surgeons made decisions with incomplete information because there was no alternative. The history and physical examination were not valued because they provided certainty; they were valued because they represented the best information available. Clinical judgment developed within the understanding that absolute diagnostic clarity was often unattainable.

Surgeons became accustomed to acting despite ambiguity. The patient with diffuse peritonitis required intervention even when the precise diagnosis remained unknown. The patient with a concerning abdominal examination often proceeded to the operating room without definitive proof of pathology. Decisions were guided by probabilities, pattern recognition, and experience rather than complete certainty.

Modern medicine has understandably sought to narrow that uncertainty. Imaging, laboratory testing, advanced monitoring, and increasingly sophisticated decision-support tools have all contributed to more precise diagnosis and risk assessment. Yet despite these advances, uncertainty has not disappeared. The patient with abdominal pain may still have equivocal imaging. The scan may be technically limited. Findings may be interpreted differently by experienced observers. Clinical deterioration continues to occur despite reassuring studies, while alarming imaging findings occasionally prove clinically insignificant. The uncertainty remains. What has changed is our response to it.

Increasingly, uncertainty is met with another test, another image, another layer of confirmation. An equivocal ultrasound leads to computed tomography. An indeterminate scan prompts repeat imaging. Additional laboratory studies are obtained. Consultations are requested. Sometimes these steps are entirely appropriate. Frequently, they improve care. Occasionally, however, they reflect a belief that sufficient information can eliminate uncertainty altogether. Repeated investigations are not without consequence. In time-sensitive surgical conditions, additional testing may delay definitive intervention when treatment is most effective. Diagnostic studies also carry their own burdens, including cost, resource utilization, and, in some cases, exposure to radiation or intravenous contrast. Although these tradeoffs are often justified, they serve as a reminder that obtaining more information is not invariably synonymous with improving care.

The bedside examination offers an uncomfortable reminder that such certainty rarely exists. Unlike imaging studies or laboratory values, the physical examination does not claim precision. It rarely provides definitive answers. Instead, it asks clinicians to interpret incomplete information and assign meaning to observations that cannot always be measured. The patient appears uncomfortable. Their breathing seems labored. They move cautiously when changing position. Their overall appearance generates concern despite reassuring objective findings.

Experienced clinicians often summarize these impressions with phrases such as "the patient looks sick" or "something doesn't seem right." Such statements can sound frustratingly subjective, particularly in an era that values measurable data. Yet these observations frequently influence important decisions. They represent the integration of countless bedside cues that resist quantification but nevertheless contribute to clinical judgment. What is commonly referred to as a physician's "gut feeling" is often the manifestation of expert pattern recognition, a cognitive process that synthesizes information more broadly than any individual test or imaging study can capture.

Perhaps this explains why the physical examination remains difficult to replace despite the superiority of modern diagnostics in many settings. Its value has never resided solely in the information it provides. Its value lies in the habits of observation and interpretation that it cultivates.

Ironically, POCUS illustrates both the strengths and tensions of contemporary medicine. POCUS is often described as an extension of the physical examination, and in many respects it is [2]. Information is gathered directly by the treating clinician at the bedside rather than obtained remotely through a formal study. Clinical assessment and diagnostic evaluation occur simultaneously. The physician remains engaged with the patient rather than detached behind a computer screen.

Yet POCUS can also reveal our increasing discomfort with uncertainty. In some circumstances, it serves not as an adjunct to clinical judgment but as a prerequisite for it. The question shifts from what the clinician observes to what the device can demonstrate. Another source of objective data is sought before committing to a decision. The technology itself is not the problem. Rather, it highlights a broader expectation that uncertainty should always be reduced before action is taken.

Surgery, however, has never been a profession of certainty. Decisions must often be made before complete information is available. The patient with mesenteric ischemia may have inconclusive imaging. The patient with a hollow viscus injury may initially appear stable. The patient with sepsis may deteriorate despite reassuring studies obtained hours earlier. Technology informs these decisions, but it does not make them. Judgment remains indispensable precisely because uncertainty persists.

This is where the decline of bedside examination becomes relevant. The concern is not the loss of specific maneuvers or nostalgia for an earlier era. Few would advocate abandoning technologies that have dramatically improved patient outcomes. The concern is that as medicine becomes increasingly adept at generating information, it may become less comfortable acting before every uncertainty has been resolved.

The physical examination teaches a different lesson. It teaches clinicians to observe carefully, interpret imperfect information, and make decisions despite ambiguity. It reinforces the understanding that medicine is not the pursuit of certainty but the management of uncertainty.

The future will undoubtedly bring more sophisticated imaging, more powerful artificial intelligence, and increasingly accurate diagnostic tools. These developments should be welcomed. They will improve our ability to diagnose disease and guide treatment. But no technology will eliminate uncertainty entirely because uncertainty is not a technological problem. It is an intrinsic feature of clinical medicine.

As our tools become increasingly powerful, the challenge is not to find certainty where none exists. It is to preserve the judgment required to act before certainty arrives.
